# Variola

**Published:** 1870-11

**Authors:** Edwin A. Carpenter

**Affiliations:** Baileyville, Illinois


### ARTICLE XXXV.

### VARIOLA.

BY EDWIN A. CARPENTER, M.D., Baileyville, Illinois.

During the past winter months we had an endemic of unmodified variola. Not a case, as far as my knowledge extends, presenting variolous symptoms, where they had ever been vacinated. The disease is ushered in with a well-pronounced chill, succeeded by considerable febrile movement, after which some slight perspiration takes place; the chills and fever continuing until the appearance of the eruption. Pain is referred to the epigastrium, with tenderness on pressure, with nausea and vomiting as the rule. Cephalolgia pain in the lumbar region and limbs, bowels constipated (usually), urine scanty and highly-colored; delirium is an occasional symptom. These are the usual symptoms of the stage of invasion. To be brief, I will not descant upon the different stages, except incidentally, as the main object in view is the treatment which differs from most medical writers on the subject. The mode of dying (where death takes place) is by asthenia. The question very naturally arises here, what produces this asthenic condition, is tissue metamorphosis sufficient to produce death? I think not. The urine being diminished in quantity, there remains in the economy, urea and uric acid, producing their toxicological effects; these, as well as all excretory products, are formed in the blood; they exist, therefore, in the blood in health, and it is their accumulation in an abnormal quantity which constitutes the morbid condition; the kidneys secreting so small a quantiny of urine, are inadequate to a proper elimination of this excrementitious substance, consequently, we have urmiæa, which is a form of blood-poisoning—sometimes called toxæmia, which enters largely into the ausation of the morbid phenomena, and consequently of great importance in its practical relations to variola.

Uræmia may be inferred, whenever in connection with its characteristic pathological effects, the secretion of the urine is suppressed, or the quantity greatly diminished. The symptoms during the stage of invasion, are indicative of congestion of the kidneys, if not of nephritis, *viz.:* pain and tenderness in the loins, pains in the abdomen, urine scanty and highly-colored, and sometimes frequent attempts at micturation. We are advised by most medical writers, to omit the use of active cathartics, but as the kidneys are inadequate to a proper elimination of urea and uric acid, what better mode of vicarious elimination than by the intestinal canal? Put the disordered kidneys at rest by causing the intestinal canal to perform their office as far as may be, and in conjunction with, elimination by the skin, their office can be performed effectually in variola, at least, all things being taken into consideration, hydragogue cathartics are a necessity for the welfare of our patient. I prefer *elaterium et podophyllum*, purging the patient freely, then, unless the pains in the head, *epigastrum*, and lumbar region are mitigated, give a hot-air bath until copious perspiration is produced, after which the recipient will feel like a different being; in all probability the affected will not be obliged to keep the recumbent posture until the suppurative stage supervenes; with the change of the eruption to pustules, there is a recurrence of the febrile movement, constituting what is known as the suppurative fever; the pulse is increased in frequency, perspiration ceases, the temperature is increased, etc.; we now have another blood-poison to deal with, caused by the absorption of pus, if skin, kidneys, and bowels are in a morbid condition, they soon eliminate the morbid matter, if not, eliminants are necessary to arouse the excretory functions of the system; where the eliminative treatment is carried out, we are rid, to a great extent, of the sequaæ of the disease, which is the result of toxæmia, *viz.:* feruncules and abcesses; at the decline of the suppurative fever quinia sulphas given as a tonic, and an eliminant, is a remedy which has proven itself peculiarly adapted to this stage of the disease.

As an ectrotic, B. T. Buckley, M.D., Freeport, Illinois, has found lime water and linseed oil (carron oil) efficient, keeping the patient in a darkened room. In my practice, I have tried a variety of vaunted remedies, but found them useless. It would not enhance the value of this article to speak of concomitant affections, alimentation, purity of atmosphere, disinfectants, etc. I wish to call the attention of the profession to the *blood-poisoning*, believing, as I do, that the retention of the effete matters causes death in variola. The foregoing is the theory on which I based my treatment in twenty cases (all I had) of the distinct and confluent varieties, ranging from 6 to 74 years of age. Not a single case resulted in death, neither was the sight or hearing impaired in a single instance.

