# Correction

**Published:** 1870-12

**Authors:** 


Correction.—In the November number of the *Examiner*, on page 676, line 17, the word *morbid* should be *normal.*

To Subscribers.—The present number completes the Eleventh Volume of the *Examiner.*

Recently, bills have been sent to all who stand on our books indebted for one year or more.

During the past year, our time has been so occupied, we have been compelled to allow the books of the *Examiner* to be kept by a clerk, and it is probable some mistakes have been made. We shall cheerfully make any corrections that our subscribers may point out. We shall commence the new volume with more reliable and steady assistance, and hope to receive the same friendly patronage as heretofore. We shall continue to make the *Examiner* strictly a periodical, devoted to the interests of the medical profession, and not an advertising sheet for booksellers and medicine manufacturers.

